# Identification of human glycosyltransferase genes expressed in erythroid cells predicts potential carbohydrate blood group loci

**DOI:** 10.1038/s41598-018-24445-5

**Published:** 2018-04-16

**Authors:** Magnus Jöud, Mattias Möller, Martin L. Olsson

**Affiliations:** 1 Hematology and Transfusion Medicine, Department of Laboratory Medicine, Lund University, Lund, Sweden; 2 Department of Clinical Immunology and Transfusion Medicine, Laboratory Medicine, Office of Medical Service, Lund, Sweden

## Abstract

Glycans are biologically important structures synthesised by glycosyltransferase (GT) enzymes. Disruptive genetic null variants in GT genes can lead to serious illness but benign phenotypes are also seen, including antigenic differences on the red blood cell (RBC) surface, giving rise to blood groups. To characterise known and potential carbohydrate blood group antigens without a known underlying gene, we searched public databases for human GT loci and investigated their variation in the 1000 Genomes Project (1000 G). We found 244 GT genes, distributed over 44 families. All but four GT genes had missense variants or other variants predicted to alter the amino acid sequence, and 149 GT genes (61%) had variants expected to cause null alleles, often associated with antigen-negative blood group phenotypes. In RNA-Seq data generated from erythroid cells, 155 GT genes were expressed at a transcript level comparable to, or higher than, known carbohydrate blood group loci. Filtering for GT genes predicted to cause a benign phenotype, a set of 30 genes remained, 16 of which had variants in 1000 G expected to result in null alleles. Our results identify potential blood group loci and could serve as a basis for characterisation of the genetic background underlying carbohydrate RBC antigens.

## Introduction

Glycosyltransferases (GTs) are the enzymes (enzyme commission [EC] 2.4) that catalyse glycosylation, resulting in a wealth of glycan variants present on glycoproteins, glycosphingolipids and proteoglycans^1^. Glycosylation is a complex form of modification of proteins and lipids, due to the large diversity in the possible structures formed by combinations of different sugar moieties and bonds, and it has been estimated that more than 50% of all proteins are glycoproteins^2^. The most prevalent type of glycosylation is the N-linked^3^, where a preformed glycan complex is bound to certain asparagine-containing motifs in the polypeptide sequence. GTs are categorised into families based on sequence similarity. The Carbohydrate-active enzymes database (CAZy)^4^ provides an updated resource for sequence-based family classifications of GTs and other carbohydrate-active enzymes by a systematic analysis of sequences deposited in Genbank. Currently, 104 GT families are recognised in CAZy, 44 of which are represented in humans.

The presence of glycans on proteins is believed to fine-tune the function of the protein, and their absence could completely abolish the function. For example, proper glycosylation is essential for synthesis and function of erythropoietin, the master regulator of erythropoiesis^5^, and for the function of immunoglobulins^6,7^. Glycans can also function as cell surface receptors in endogenous processes^8^ and host-pathogen interactions^9,10^. The importance of proper glycosylation is also noted in the rare group of disorders collectively known as congenital disorders of glycosylation (CDG)^11^. In CDG, genetic variation resulting in inactivation of genes responsible for glycan biosynthesis and glycan metabolism causes a heterogeneous group of phenotypes. Defects of core GTs generally result in more severe phenotypes than in more terminal GTs^11^. Whilst the prevalence of CDG is largely unknown, it has been estimated in Europe to be 0.1–0.5/100,000^12^, but is generally believed to be under-reported due to the heterogeneity of symptoms, making it difficult to identify affected patients^11,13^. The most common subtype of CDG is PMM2-CDG, representing about 68% of the recorded CDG cases^12^. It is caused by various disrupting mutations in the phosphomannomutase 2 gene (*PMM2*) and the associated symptoms are broad and highly variable^14^.

Deficiencies in GT function do not always result in a disease phenotype, but could still be of clinical importance. Genetic variation in GT genes can result in both qualitative and quantitative differences in glycans expressed on the red blood cell (RBC) surface. These differences can be immunogenic and effectively act as a barrier in transfusion and transplantation. Out of the 36 blood group systems recognised by the International Society of Blood Transfusion (ISBT)^15^, seven (ABO, P1PK, Lewis, H, I, Globoside and FORS) are carbohydrate-based^16^ with ABO being the first described and most well-known. In fact, homozygosity or compound heterozygosity for null alleles at known blood group loci often underlies antigen-negative blood group phenotypes, for example in ABO where the c.261delG polymorphism in ABO*O.*01* alleles causes a frame shift in the amino acid sequence resulting in a non-functional enzyme^17^. In addition, the high-frequency antigens Sd^a^, LKE and i (ISBT no. 901012, 209003 and 207002, respectively), are known to be carried on glycans but their underlying genetic backgrounds have not been fully explained, i.e. they are orphan blood groups. The consequence is that genotypic phenotype prediction is not yet possible. Previous work has suggested *B4GALNT2* as the gene responsible for Sd^a^ synthesis^18,19^, however, it has not yet been shown that variation in *B4GALNT2* actually gives rise to the Sd(a‒) phenotype. The genetic background of LKE is more elusive but a mutation in *B3GALT5* has been linked to weak expression of the antigen in African Americans^20^. Thus, the genetic basis of the LKE-negative phenotype has yet to be determined. Whilst the gene underlying the I antigen and mutations responsible for the I-negative phenotype have been elucidated^21^, the genetic basis of the expression of its precursor, i, remains unexplained.

We have previously dissected the genetic variation in all 43 blood group genes underlying expression of all human blood group systems recognised by ISBT, including those giving rise to carbohydrate histo-blood groups^22^. In this study, we aim to identify all expressed human GT genes and assess their potential as blood group gene candidates by investigating the genetic variation in these genes in data generated by the 1000 Genomes Project (1000 G), in combination with their erythroid expression pattern^23^. We further examine the expression of GT genes in RBCs, with the aim of finding candidate genes underlying the expression of orphan and emerging carbohydrate blood group antigens.

## Results

**Figure 1:**
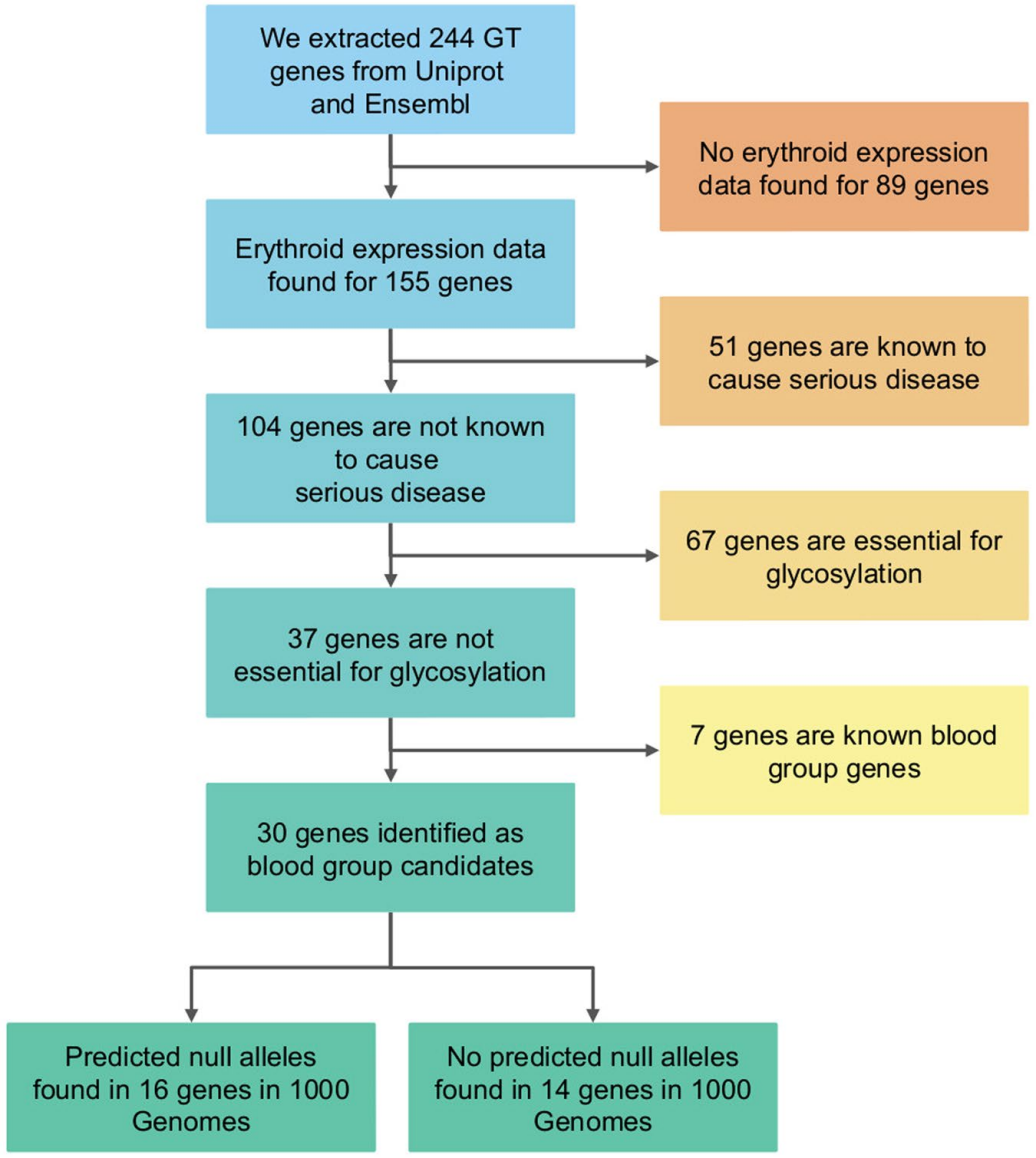
Summary of the analysis work flow. We searched for expressed human GTs in public databases and found 244 GT genes matching the search criteria. The genetic variants called by 1000 G within the limits of these genes were collected and annotated. We filtered the genes in a stepwise algorithm to find dispensable genes expressed in erythroid cells. The final set of genes are the candidate blood group genes, some of which have variants predicted to result in null alleles.

**Figure 2:**
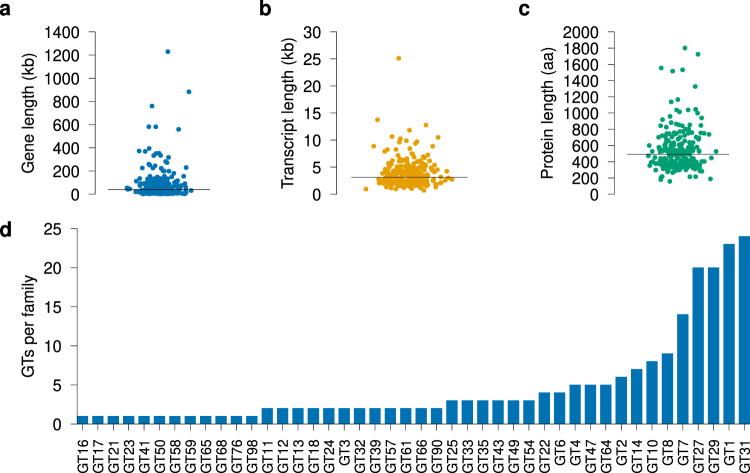
Descriptive data for the identified human GT genes and their transcripts and predicted protein products. **(a)** The lengths of the GT genes varied considerably, with a median length of 40,799 base pairs (bp) (Supplementary Table 1). The total length of all GT genes was 20,563,185 bp, or 0.7% of the lengths of chromosomes 1–22 and chromosome X in GRCh38 combined. **(b)** Distribution of lengths for the canonical transcript for each gene and **(c)** length distribution for the predicted protein product for each of the transcripts in **(b)**. The lengths of the canonical transcripts were shorter, with a median length of 3,134 bp and a median protein product length of 492 amino acids (aa). **(d)** Distribution of GT families. Out of the 244 GTs found, 199 (82%) were annotated in Uniprot and the CAZy database as members of at least one GT family (Supplementary Table 1). These GTs were distributed over 44 distinct GT families, GT31 being the most abundant family. The black bar in **(a**–**c)** represent the median value for each plot.

The study workflow is summarised in Fig. 1. We searched the UniProt^24^ database for all human GTs with predicted expression on protein level. Following these searches, we found 244 genes, representing 44 GT families, matching the search criteria (Fig. 2, Supplementary Table 1) and annotated these with data from Ensembl^25^. We did not find any additional GTs in the CAZy database^4^.

### Distribution and consequences of genetic variants in GTs

**Figure 3:**
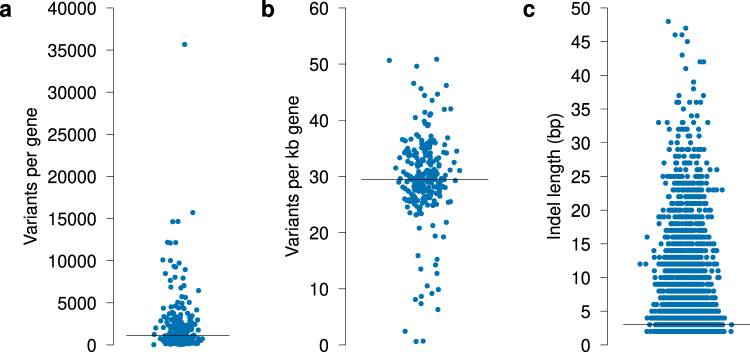
Genetic variation in human GTs. We extracted all genetic variants with rs numbers called by 1000 G. (**a**) Number of variants per GT gene. We found a large variation in the number of variants per GT locus, with an almost 1000-fold difference between the highest and the lowest. (**b**) Number of variants per GT gene, normalised by gene length. (**c**) Distribution of the length of indels in the 1000 G data. Whilst most indels were short (median 3 bp), indels up to 48 bp in length were present. The black bar in the graphs represents the median value.

To investigate the genetic variation in GT genes, we used the data provided by 1000 G^23^. In total, 550,275 variants were called in 1000 G within the genomic limits of GT genes as defined in the Ensembl database, 543,040 of which were single nucleotide variants (SNVs). The remaining 7,235 variants were insertions or deletions (indels) with a median length of 3 base pairs (bp) (range, 2–48) (Fig. 3).

We found large differences in the number of variants per locus, with a median of 1,106 (range, 38–35,673) (Fig. 3, Supplementary Table 1). Whilst normalisation of the number of variants by gene length decreased this difference, a large variation in the normalised variation frequency persisted (median 29.5, range 0.61–50.9) (Fig. 3). The most variable GT genes were *ALG1L*, *APRT* and *RFNG* with 50.9, 50.7 and 49.6 variants/kb, respectively. *MGAT4C*, *B3GALNT2* and *ST6GAL1* were considerably more preserved, showing the least variation with 0.61, 0.70 and 2.44 variants/kb, respectively. Disruptive variants in *B3GALNT2* are strongly associated with congenital muscular dystrophy-dystroglycanopathy with brain and eye anomalies (type A11; MDDGA11)^26^. Disease associations for *MGAT4C* and *ST6GAL1* are weaker, but genetic variation in these genes has been implicated in prostate cancer^27^ and type 2 diabetes^28^, respectively.

Next, we classified the predicted consequences of the variants using the Variants Effects Predictor tool (VEP)^29^. The vast majority of all variants (98.7%) were located outside exons and splice regions of canonical transcripts, or were synonymous (Table 1). All but four GTs (*ST6GAL1*, *HPRT1*, *MGAT4C* and *OGT*) had missense variants or other variants predicted to result in a change in amino acid sequence (Supplementary Table 1). The number of variants classified by VEP as having high impact, generally predicted to result in null alleles, i.e. resulting in a non-functional gene product, was 329 (0.6%). These high impact variants, including frameshifts, splice donor or acceptor mutations, lost start or stop codons, or stop gained variants, were distributed over 149 GTs (61%), with *FUT2* having the most (n = 9). Among the GTs with the highest number of amino acid-altering variants, we found the *AGL* and *XYLT1* genes, known to cause the glycogen storage disease type III^30^ and Desbuquois dysplasia type 2^31^, respectively.

**Table 1:** Sequence ontology consequence terms for genetic variants in human GT genes.

Sequence Ontology term	n
upstream_gene_variant	43,719
downstream_gene_variant	23,128
intron_variant	455,036
splice_region_variant, intron_variant	603
frameshift_variant, splice_region_variant, intron_variant	1
exon_variant	
3_prime_UTR_variant	13,580
missense_variant	6,904
synonymous_variant	4,146
5_prime_UTR_variant	2,538
stop_gained	205
missense_variant, splice_region_variant	153
splice_region_variant, synonymous_variant	93
splice_donor_variant	38
splice_acceptor_variant	34
frameshift_variant	27
splice_region_variant, 5_prime_UTR_variant	19
inframe_deletion	13
start_lost	12
stop_retained_variant	8
stop_lost	6
inframe_insertion	4
stop_gained, splice_region_variant	3
coding_sequence_variant	1
start_lost, 5_prime_UTR_variant	1
frameshift_variant, splice_region_variant	1
protein_altering_variant	1
frameshift_variant, stop_retained_variant	1

### Most GT haplotypes are unique to a single superpopulation in 1000 G

Since the 1000 G genotype data is phased, we could combine the distinct variants into predicted haplotypes. We found 8,289 unique protein haplotypes in GT genes, with a median of 27 haplotypes per GT (range, 1–209) (Supplementary Table 1). The four GTs without any amino acid-altering variants had only a single protein haplotype each (*ST6GAL1*, *HPRT1*, *MGAT4C* and *OGT*). Out of all protein haplotypes, 6,589 (79.5%) were unique to a single continental superpopulation represented in 1000 G (AFR, AMR, EAS, EUR, SAS)^23^, a proportion slightly higher than a control set of random length-matched genes (78.0%; *p* = 2.5 × 10^−16^, χ^2^-test).

### A majority of GT genes are expressed in RBCs

**Figure 4:**
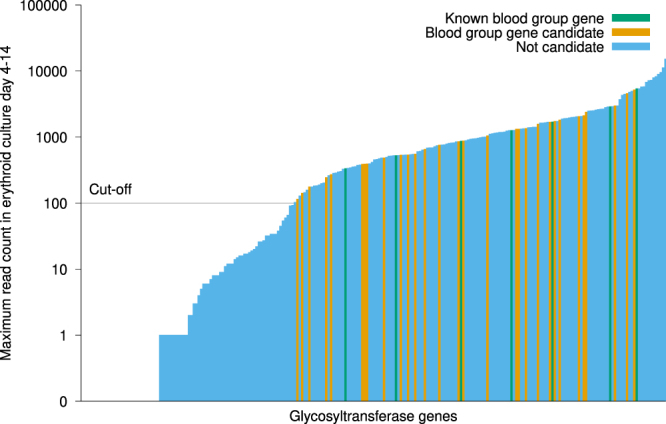
Gene expression for GT genes in erythroid cells. We used RNA-Seq data generated by Shi *et al*.^32^. to find GTs that are expressed in erythroid cells. Using a cut-off determined by the expression levels of blood group-related GT genes known to be expressed in RBCs (*A4GALT*, *ABO*, *ART4*, *B3GALNT1*, *FUT1*, *GBGT1* and *GCNT2*), we found that a majority of GT genes (n = 155) are expressed in RBCs at a level above the cut-off. The genes that remained after filtering for blood group gene candidates and the blood group-related GT genes are indicated in contrasting colours. The cut-off value used is indicated by a horizontal line.

To evaluate the possibility of expression of GTs in RBCs, we used a RNA-Seq data set generated in CD34+ cells cultured under conditions favouring erythropoietic development^32^. We limited the list of candidate genes to those with an expression level similar to or higher than the known blood group-related GT genes, excluding *FUT2* and *FUT3* (known not to be expressed endogenously in erythroid cells). We found 155 GT genes (64%) to be expressed at this level (Fig. 4, Supplementary Table 2). Eighty-one GT genes were expressed below this level, whilst expression data was missing for the remaining eight.

To further validate this set of genes, we investigated the presence of ChIP-Seq peaks for the erythroid transcription factor GATA1 in the Encyclopedia of DNA Elements (ENCODE)^33^ data, overlapping the limits of these genes. We found at least one GATA1 ChIP-Seq peak in 85 of the 155 GT genes expressed in RBCs, as compared to 20 in the other 89 genes (Supplementary Table 3), in any of the erythroid cell lines K562, PBDE or PBDEFetal. A significant difference (*p* = 1.7 × 10^−6^, χ^2^-test), this enrichment was mainly driven by the GATA1 ChIP-Seq peaks in PBDE (81 *vs*. 19; *p* = 4.4 × 10^−6^, χ^2^-test) and K562 (28 *vs*. 4; *p* = 0.005, χ^2^-test) while there was no difference in the PBDEFetal cell line (17 *vs*. 9; *p* = 0.99, χ^2^-test) (Supplementary Table 3). We found GATA1 ChIP-Seq peaks in all but one (*B3GALNT1*) of the known blood group-related genes.

### Selection of potential blood group genes

To identify additional potential blood group-related GT genes, we further refined the list of GT genes expressed in erythroid cells in several steps (Fig. 1). First, we removed all genes that are known to be disease-causing as indicated by entries in the Orphanet database, a compilation of rare diseases and their underlying genetic backgrounds (http://www.orpha.net/). The rationale behind this was that the presence of irregular, yet unidentified, but likely naturally-occurring blood group antibodies in a rare patient group would be a well-known phenomenon. Second, we removed all other GT genes predicted to be essential for glycosylation at large, including core GPI-anchor, O- and N-glycosylation genes, as indicated by database searches. Finally, we removed the known blood group-related genes, all of which were still remaining at this last step.

**Figure 5:**
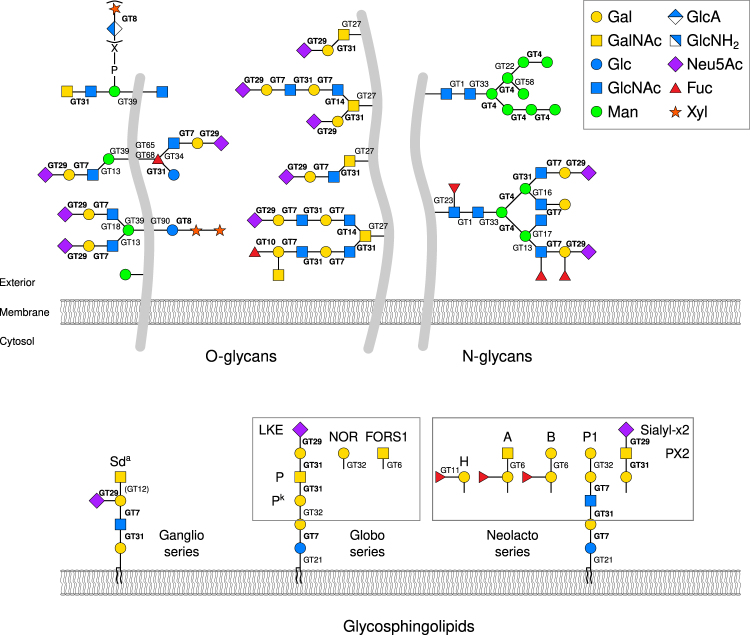
Selected glycan types expressed on RBCs, including some known blood group antigens. We determined a set of genes expressed in RBCs and with predicted benign impact (Table 2). The disruption of these genes could potentially lead to alterations in cell surface antigens and incompatibility in transfusion and transplantation. Models of a selected subset of O- and N-glycans and glycosphingolipids with predicted expression on the RBC surface are shown. Indicated are the GT families that are present in the list of GTs expressed in RBCs (Supplementary Table 2). Furthermore, the GT families highlighted in bold font all have members listed as candidate genes in Table 2. Names of known glycan blood group antigens are indicated. Sialyl-x2 is a suggested but not yet officially acknowledged blood group^40^. The Sd^a^, A, B, and H antigens are known to be expressed also on glycoproteins but are shown here only on glycosphingolipids for reasons of graphic clarity. This figure was modified and updated from Hansen *et al*.^13^.

Using this filtering strategy, we found 30 GT genes that were expressed in RBCs (18 with GATA1 ChIP-Seq peaks) and where a null allele was predicted to result in a non-pathogenic variant and thereby a benign phenotype (Table 2). Notably, 16 of these had variants in 1000 G predicted to cause null alleles (Table 2, Supplementary Table 4). Homozygosity or compound heterozygosity for these alleles could result in a null phenotype and potentially the absence of a glycan cell surface antigen (Fig. 5). Furthermore, 29 genes (excluding *ST6GAL1*) had variants classified as having moderate impact. Among these variants, we found variants classified to be damaging and deleterious by the PolyPhen-2 and SIFT tools, respectively, potentially disrupting protein function. To find even more rare null alleles, not represented in 1000 G, we searched the Genome Aggregation Database (gnomAD)^34^ for loss of function variants in the candidate genes, and found that all 30 candidate genes had such variants in gnomAD (Table 2, Supplementary Table 4).

**Table 2:** Candidate GT genes expressed in RBCs with a benign predicted impact.

Gene name	GT family	1000 G high impact variants		1000 G moderate impact variants		gnomAD loss of function variants
		Total (n)	Allele frequency (%)	Total (n)	PolyPhen-2 damaging and SIFT deleterious variants (n)	Total (n)
*B3GNT2*	GT31	0	0	15	2	4
*B3GNT9*	GT31	0	0	17	8	11
*B3GNTL1*	GT2	5	0.30	37	18	50
*B4GALT2*	GT7	0	0	20	5	8
*B4GALT3*	GT7	0	0	19	6	15
*B4GALT4*	GT7	3	0.06	11	1	19
*B4GALT6*	GT7	0	0	16	2	9
*DPY19L1*	unknown	1	0.02	21	4	14
*DPY19L3*	GT98	0	0	32	9	42
*DPY19L4*	unknown	4	0.20	39	10	81
*FUT4*	GT10	0	0.06	22	6	33
*FUT7*	GT10	0	0.08	37	15	13
*FUT10*	GT10	3	0	36	7	40
*FUT11*	GT10	2	0	27	13	39
*GCNT1*	GT14	1	0.02	15	4	20
*GLT8D1*	GT8	1	0.14	15	5	31
*GTDC1*	GT4	1	0.02	37	12	35
*GXYLT1*	GT8	0	0	15	2	26
*KDELC1*	GT90	0	0	30	10	31
*ST3GAL1*	GT29	0	0	21	0	6
*ST3GAL2*	GT29	1	0.02	18	0	6
*ST3GAL4*	GT29	1	0.02	13	2	8
*ST3GAL6*	GT29	1	0.04	15	4	31
*ST6GAL1*	GT29	0	0	0	0	12
*ST6GAL2*	GT29	0	0	31	0	21
*ST6GALNAC1*	GT29	1	0.04	57	0	46
*ST6GALNAC4*	GT29	1	0.02	19	0	16
*ST6GALNAC6*	GT29	2	0.06	22	0	15
*ST8SIA4*	GT29	0	0	7	0	10
*ST8SIA6*	GT29	3	0.06	23	10	23

## Discussion

We investigated human GT loci and characterised the variation in these genes using data generated by 1000 G. Moreover, we identified GT genes expressed in RBCs, where genetic variations causing null phenotypes could form the bases of hitherto unrecognised blood group systems. We present a list of prime candidates for further exploration in the ongoing search for genetic homes for orphan and emerging blood group antigens of carbohydrate nature.

RNA-Seq is a sensitive and unbiased method for transcriptome analysis, with excellent dynamic range. Using such a dataset, we found that a majority of GTs are expressed in erythroid cells at different levels. Even so, the possibility of false negative results among the null- or near null-expressing GT cannot be excluded. Although it can be expected that novel blood group genes would be expressed at a level equivalent to, or higher than, those in existing blood group systems in RBCs, we cannot rule out the possibility of novel blood group genes having lower expression. It is also possible that the expression is transiently higher earlier in erythropoiesis. We also acknowledge the fact that some blood group antigens, such as different Lewis antigens, are carried on glycosphingolipids adsorbed onto the RBC membrane, thus being synthesised in other cell types. Accordingly, such GT genes would not be identified in data generated in erythroid cells. This may well be the reason that we did not detect any erythroid expression of the orphan Sd^a^ candidate gene *B4GALNT2* in this dataset. Consistent with this finding, *B4GALNT2* did not have ChIP-Seq peaks for the erythroid transcription factor GATA1, which was found in all but one of the known blood group-related GT genes. This may also be consistent with the fact that Sd^a^ antigen is found in both human urine, saliva, meconium and to some degree also in plasma^35^. Clearly, further studies on the genetic basis of the Sd(a‒) phenotype are required.

Homozygosity or compound heterozygosity for disruptive variants is known to cause null alleles, which is the reason we used this as one of our selection criteria to home in on the most likely blood group GT gene candidates. However, other types of variation can also lead to dysfunction of the enzymatic activity of GTs. Point mutations at critical residues in the GT can be disruptive or alter the function of the enzyme, as exemplified by ABO*O.*02*, an infrequent form of null (blood group O) alleles in the ABO system due to c.802 G>A (p.Gly268Arg). This may be difficult to predict computationally and the effect of these variants requires further study. Furthermore, there is a possibility that structural variation in GTs, not captured here, might contribute to the frequency of null alleles beside SNVs and smaller indels. Taken together, though, missense variants and SNVs are quite uncommon as the reason underlying blood group negative carbohydrate phenotypes.

The list of candidate genes resulting from this study could serve as a basis for investigations into known and novel blood group systems, however, determining whether the glycan products of any of these GTs are actually present on the cell surface of RBCs is beyond the scope of this study.

Interestingly, among the candidate GT loci we found four genes from the GT29 family with α-2,3-sialyltransferase activity (*ST3GAL1*, *ST3GAL2*, *ST3GAL4*, *ST3GAL6*). This type of activity is predicted to be necessary for the synthesis of the sialylated orphan LKE blood group antigen^16^. We found that all these candidates had variants predicted to cause a null allele. It has previously been suggested that missense variants in *B3GALT5*, encoding the LKE precursor galactosylgloboside, give rise to the LKE+ weak phenotype^20^. However, we did not find *B3GALT5* to be expressed in RBCs, which may imply that inability to synthesize the terminal linkage between galactosylgloboside (Gb5) and sialic acid may be the crucial defect leading to LKE-negativity. Notably, the LKE-negative phenotype is quite uncommon (1–2%) whilst a LKE-weak phenotype has a frequency at 10–20%^16^. The former may represent homozygosity for a null allele at the implicated locus whilst the latter may be due to heterozygosity. Thus, the short-listed candidate sialyltransferases should be of interest to investigate in LKE-negative and LKE-weak individuals.

Besides Sd^a^ and LKE, the enigmatic i blood group antigen belongs to the category of orphan carbohydrate blood groups, already acknowledged by ISBT but still not part of a blood group system. Since this antigen appears to be an epitope internally located in the glycan chain and present in the absence of functional *GCNT2*-encoded enzyme, it is less clear what type of GT is lacking in individuals negative for the i antigen, or if such individuals even exist.

In addition to the three orphan antigens mentioned above, 38 of the currently acknowledged blood group antigens have unknown molecular carriers and therefore no gene known to govern their expression. Many of these are expected to be protein-based antigens since only seven of the current 36 blood group systems are carbohydrate-based but it is fully possible that some are glycans. Furthermore, as exemplified by the recent discovery of the FORS blood group system^36^, emerging blood groups not previously acknowledged by ISBT or even known to exist on human RBCs at all, can be carried of glycans. The role of the highlighted 30 final candidate blood group GTs in the expression of orphan and emerging carbohydrate blood groups remains to be determined, but we anticipate that the results of this study will provide a panel of interesting candidate genes for testing by investigators in specialized immunohematological reference laboratories and research institutions.

## Methods

### Protein, gene and transcript data retrieval

We searched UniProt^24^ (release 2017_08) for human GTs using the search term *(“ec:2*.*4*.*-*.*-” OR “cazy:GT”) AND organism:“Homo sapiens (Human) [9606]” AND reviewed:yes* using the UniProt representational state transfers (REST) application programming interface (API). In each search result, we extracted the first cross-referenced Ensembl transcript identifier and used it to retrieve transcript and gene data from the Ensembl database (release 90). For two Uniprot entries, corresponding to genes *ABO* and *B3GALT4*, there was no Ensembl cross-reference annotated (*ABO*), or the first cross-reference referred to an alternative gene assembly (*B3GALT4*). We used manually specified transcript identifiers for these two entries (Supplementary Table 5). One search result, UniProt entry A8MXE2, was annotated by Ensembl as a pseudogene and was discarded from further analysis.

We then downloaded transcript and cross-linked gene data for each predicted GT using the Ensembl REST API^37^ (release 90). The gene limits defined by the Ensembl data correspond to the outermost start and end coordinates of all transcripts of the gene. Three genes (*GCNT6*, *DPY19L2P1* and *DPY19L2P2*) were not annotated as protein coding and were discarded from further analysis. In the gene data, we identified the canonical transcript as the basis of further transcript analysis. For two entries, *ABO* and *B3GALT4*, no canonical transcript was annotated and instead we used the same manually specified transcript identifier as in the UniProt search (Supplementary Table 5).

### Variant data retrieval and haplotype determination

We used the phase 3 release of 1000 G^23^, downloaded from ftp://ftp.1000genomes.ebi.ac.uk/vol1/ftp/release/20130502/, for extraction of haplotypes and as the basis for the selection of variants. We used the Ensembl REST API to convert genomic start and end coordinates from GRCh38 to GRCh37 (used by 1000 G), and Tabix^38^ to extract the variants located within these positions from the VCF files. All single nucleotide variants, insertions and deletions with rs numbers were extracted. From the generated data subset, we extracted haplotype information based on the phase information for each genotype call. We downloaded variant consequence data for all extracted genetic variants from the Ensembl variant effect predictor (VEP)^29^ REST API endpoint (release 90). Finally, the Genome Aggregation Database (gnomAD)^34^ data browser at http://gnomad.broadinstitute.org/ (version r2.0.2) was used to locate additional loss of function variants for the candidate genes.

To compare the proportions of protein haplotypes unique to any population in 1000 G, one random length-matched gene (GT gene length ± 2.5%) for every GT gene was retrieved from Ensembl (release 90). For all protein haplotypes in the 1000 G, we then counted those unique to a single superpopulation in 1000 G. The number of unique haplotypes and the total haplotype count were then compared to the corresponding numbers in GTs.

### Expression of GT genes in erythroid cells

To determine the expression of GT genes in erythroid cells, we used data provided by Shi *et al*.^32^, where they performed RNA sequencing on human CD34^+^ cells at four time points during differentiation from earliest into more mature erythroid cell stages. We downloaded RNA-Seq data for each GT from the author’s website (http://guanlab.ccmb.med.umich.edu/data/Shi_L_Developmental). We defined a cut-off level for read count at 100 to filter out background noise in the data. All genes with a read count ≥ 100 at any point of measurement (day 4, 8, 11 or 14) were considered expressed in RBCs. This included all blood group-related GT genes known to be expressed in RBCs (*A4GALT*, *ABO*, *ART4*, *B3GALNT1*, *FUT1*, *GBGT1* and *GCNT2*), excluding *FUT2* and *FUT3*, known not to be expressed endogenously in RBCs.

### Locations of GATA1 ChIP-Seq peaks

We downloaded locations of GATA1 ChIP-Seq peaks generated by the ENCODE project^33^ from UCSC (table wgEncodeRegTfbsClusteredWithCellsV3). The input data was filtered to include only GATA1 peaks in the erythroleukemic cell line K562, peripheral blood-derived erythroblasts (PBDE) or PBDEs in human fetal liver (PBDEFetal). The start and end positions were then lifted from hg19 to hg38 coordinates using the UCSC liftOver tool with the hg19ToHg38 chain file.

### Prioritisation of candidate blood group genes

We used Orphanet (http://www.orpha.net/), Kegg glycan^39^ and PubMed as resources for information on the dispensability of GT genes expressed in RBCs. Any GT gene where dysfunction was noted to result in a disease phenotype was removed from the list of candidates. Furthermore, we removed all the genes functioning proximally to the genes removed in the first step, as indicated by glycan synthesis pathway maps.

### Statistical analysis

We used Python with the Pandas (http://pandas.pydata.org/) and Scipy packages (http://www.scipy.org/) for statistical analysis. We considered two-sided *p*-values < 0.05 to be statistically significant.

### Data availability

The datasets generated and/or analysed during the current study are available from the corresponding author on reasonable request. Source code for the programs used to generate the data is available from https://bitbucket.org/mjoud/gt/.

## Electronic supplementary material

Supplementary Table 1

Supplementary Table 2

Supplementary Table 3

Supplementary Table 4

Supplementary Table 5
